# Carbon Quantum Dots as a Luminescent Platform for Photoswitchable Bioactive Hybrids: Tuning Butyrylcholinesterase Inhibition Through Functional Group Engineering

**DOI:** 10.3390/nano16171066

**Published:** 2026-08-27

**Authors:** Ilya Kolesnikov, Gulia Bikbaeva, Anastasia Egorova, Anna Pilip, Aleksandra Levshakova, Kirill Laptinskiy, Alexey Vervald, Tatiana Dolenko, Xiaojun Han, Alina A. Manshina

**Affiliations:** 1 St. Petersburg State University, 7–9 Universitetskaya Embankment, St. Petersburg 199034, Russia; ie.kolesnikov@gmail.com (I.K.); st086467@student.spbu.ru (G.B.); diekerze54@gmail.com (A.E.); anyta_273@mail.ru (A.P.); sashkeens@gmail.com (A.L.); 2 St. Petersburg State Technology Institute (Technical University), 26, Moskovski Ave., St. Petersburg 190013, Russia; 3 St. Petersburg Federal Research Center of the Russian Academy of Sciences (SPC RAS), Scientific Research Centre for Ecological Safety of the Russian Academy of Sciences, Corpusnaya 18, St. Petersburg 197110, Russia; 4 M.V. Lomonosov Moscow State University, Moscow 119991, Russia; laptinskiy@physics.msu.ru (K.L.); alexey.vervald@physics.msu.ru (A.V.); tdolenko@mail.ru (T.D.); 5 Heilongjiang Provincial Joint Laboratory of Molecular Science (International Cooperation), School of Chemistry and Chemical Engineering, Harbin Institute of Technology, Harbin 150001, China

**Keywords:** multifunctional materials, carbon quantum dots, butyrylcholinesterase inhibition, luminescence, photoactivation of bioactivity, photopharmacology

## Abstract

Light-responsive materials enabling external modulation of bioactivity and spatial control are highly requested for photopharmacology—a booming research area of modern medicine. We present organo-inorganic hybrids of photoswitchable, bioactive symmetric diamine-phosphine oxides conjugated with luminescent carbon quantum dots (CQDs). The phosphonate compound was found to undergo *Z*-*E* isomerization upon 266 nm laser irradiation and exhibit butyrylcholinesterase (BChE) inhibition that increases twofold (15–30%) after photoconversion. Hybrids were fabricated via physical adsorption and chemisorption using different CQD surface groups, and characterized by UV-Vis, luminescence, and FTIR spectroscopy, confirming hybrid formation and retention of functional properties. In both binding modes, the molecules retained photoswitching capability despite steric constraints. All hybrids displayed orthogonal functions: luminescence (excitation at 350 nm) and photomodulation of BChE inhibition (at 266 nm). Remarkably, the binding mode dictated the bioactivity window—chemisorbed hybrids showed narrow 1.5-fold modulation, whereas physisorbed hybrids exhibited ultra-wide >10-fold modulation. This tunable responsiveness, achieved simply by altering the binding mode, demonstrates the exceptional potential of this hybrid design strategy for developing photoswitchable materials with tailored photopharmacological performance.

## 1. Introduction

As the demand for multifunctional materials continues to grow, hybrid nanostructures have emerged as a central focus of research, driving innovations across multiple disciplines and paving the way for next-generation technologies [1,2]. Within this vast family, photoswitchable hybrid nanomaterials have attracted particular interest. These hybrids are conjugates of a nanocarrier and photoswitchable molecules capable of undergoing conformational transitions upon light irradiation. The most extensively studied photoswitches include azobenzenes, spiropyrans/merocyanines, and diarylethenes, whose common feature is the reversible transition between isomeric forms induced by optical radiation [3,4]. As nanocarriers, a wide variety of materials have been successfully demonstrated, including plasmonic nanoparticles [5], rare-earth-doped luminescent nanoparticles [6], various carbon nanomaterials such as fullerenes, graphene, and carbon nanotubes [7], magnetic nanoparticles [8], semiconductor quantum dots [9], etc. The applications of photoswitchable hybrids span a variety of energy storage devices, nanoelectronics, sensors, and high-resolution bioimaging [7]. Although the reported progress is substantial, the full design space of photo-responsive systems, including synergistic combinations of photoswitchable and functional properties, remains largely unexplored. Thus, further research to unlock functionalities far beyond current demonstrations is highly promising.

Considering the fundamental basis underlying the diversity of photoswitchable hybrid materials, the key feature is the ability of the photoswitchable molecule to undergo conformational transitions under steric constraints imposed by conjugation with the functional nanoparticle carrier [10,11]. In this context, photoswitching efficiency is governed by the degree and nature of the molecule-carrier binding, as well as the stability of the hybrid in each isomeric form. Given the vast number of possible photoswitchable molecules and nanocarriers, investigating this aspect demands extensive analytical effort and further systematic organization.

Among the various nanocarrier options, carbon nanostructures stand out as particularly promising due to their broad functionalization capabilities. Within this class, carbon quantum dots (CQDs) are especially notable, as they combine wide-ranging surface functionalization potential with an important practical property—luminescence. Moreover, CQDs’ intrinsic biocompatibility, low toxicity, and tunable optical properties make them ideal candidates for biomedical applications, including bioimaging, drug delivery, and theranostics [12,13,14]. Thus, CQDs serve as convenient model systems that bridge fundamental surface chemistry and practical functionality. Currently, photoswitchable hybrids based on CQDs have been reported for several types of photoswitches, demonstrating photo-switchable luminescence as their primary functional property [15,16,17,18].

To expand the applicability of such hybrids, additional functionality would be promising. Notably, we have previously identified families of photoswitchable phosphonates that also exhibit pronounced bioactivity—specifically, the ability to inhibit butyrylcholinesterase (BChE)—with the bioactivity level depending on the conformational state of the molecule [19,20,21]. Moreover, our prior research has demonstrated that these molecular properties (photoswitchable BChE inhibition) are preserved upon conjugation with various nanocarriers, including fullerenes, oxide nanoparticles doped with rare-earth ions, and carbon quantum dots [22,23,24]. A pronounced effect of the nanocarrier on both the photoswitching behavior and bioactive properties of the resulting hybrids was observed. However, the different nature of nanocarriers and the diverse modes of their interaction with the photoswitchable molecules have precluded a systematic analysis of this effect.

The present study, therefore, aims to investigate the relationship between the functional groups of carbon quantum dots and the photoswitching ability of the conjugated molecules, as well as the impact on the bioactivity window of these hybrids before and after laser irradiation. As a molecule providing both photoswitchable and BChE inhibition functions, the symmetrical (2-chloro-2 phenylethenyl)(diamine)phosphine oxide (PP) was chosen, whereas a series of CQDs served both luminescence and surface group diversity. Thus, our research integrates the demonstration of enhanced hybrid functions (photoswitching, butyrylcholinesterase inhibition, and luminescence) with an analysis of how the binding mode between the hybrid components affects these functional outcomes.

## 2. Materials and Methods

### 2.1. Materials for Phosphonate Synthesis

The following reagents were used in the work: carbon tetrachloride (Vekton, St. Petersburg, Russia), triethylamine (Vekton), tetrahydrofuran (Vekton), and methylpiperazine (Sigma-Aldrich, Singapore). All chemicals were commercially available as chemical grade.

#### Phosphonate Synthesis

In a round-bottomed flask equipped with a magnetic stirrer, 2-phenylethynylphosphonic acid dichloride (1) (0.005 mol) was dissolved in 25 mL of carbon tetrachloride. A mixture of corresponding amine (0.01 mol) and triethylamine (0.0125 mol) in 5 mL of carbon tetrachloride was slowly added dropwise with constant stirring and cooling. The reaction mass was constantly stirred for 13–26 h until the initial dichloroanhydryl completely disappeared (monitored by ^31^P NMR spectra). The solvent was distilled off from the reaction mixture on a rotary evaporator. The mixture was then treated with 5 × 5 mL of tetrahydrofuran and filtered through a paper filter. The solvent was distilled off from the filtrate on a rotary evaporator. The resulting substances were yellow oils, which were subsequently recrystallized in a 1% hexane:benzene solution. The final product of phosphonate (PP) was isolated as white crystals with yields of up to 96%. The reactions were carried out under a nitrogen atmosphere. The reaction progress was monitored by a ^31^P NMR spectroscopy method.

### 2.2. Synthesis of CQD

#### 2.2.1. Materials for CQD Synthesis

Citric acid (CA, Sigma Aldrich), ethylenediamine (EDA, Acros Organics, Geel, Belgium) and thiourea (TU, Sigma Aldrich) were used as precursors for CQD synthesis. Bidistilled deionized water (conductivity of 0.055 μSm/cm, Millipore Milli-Q water purification system) was used as a solvent in all experiments.

#### 2.2.2. Synthesis of CQDs

CQD1 was synthesized using aqueous solutions of EDA and CA in a molar ratio of EDA:CA of 2:1 (0.2 M:0.1 M). The precursor mixture (10 mL) was poured into a Teflon container of a steel autoclave, which was then placed in a muffle furnace (Sputnik, Kirov, Russia) and kept there for 3 h at a temperature of 200 °C. The synthesis time was counted from the moment the furnace reached the required temperature; the heating rate was ~25 °C/min. After the synthesis time had elapsed, the autoclaves were removed from the furnace and cooled naturally to room temperature.

CQD2 was obtained similarly: as a result of the reaction of EDA and CA in a ratio of EDA:CA of 20:1 (2 M:0.1 M) for 3 h at a temperature of 140 °C. In this way, nitrogen-doped CQD1 and CQD2 were synthesized.

CQD3 samples were synthesized from TU and CA with a TU:CA ratio of 1:1 (0.1 M:0.1 M) for 4 h at 160 °C. As a result, CQD3 doped with nitrogen and sulfur atoms was obtained. In our studies, the specific conditions for the synthesis of CQD3 were taken from the article [25], in which the optical properties of such CQDs were studied depending on the synthesis conditions and solvents.

After synthesis, the CQDs were purified from other reaction products. A membrane syringe filter with a pore size of 0.22 μm was used to remove large fractions. Then, the CQDs were dialyzed using a dialysis bag with a pore size of 500 Da to remove non-interacted precursor molecules. The concentration of particles in the resulting solutions, measured by evaporation and weighing of a portion of the samples, was 23, 38, and 21 mg/mL for CQD1, CQD2, and CQD3, respectively.

### 2.3. Synthesis of Hybrids

Hybrid CQD+PP nanostructures were synthesized by combining an aqueous colloidal suspension of each CQD with 1,1′-([(Z)-2-chloro-2-phenylethinyl]phosphoryl)dimethylpiperazine (PP) according to previously reported protocol [23]. Specifically, 3.82 mg of PP was added to 1 mL of a 1 mg/mL aqueous suspension of CQDs to achieve a PP concentration of 10^−2^ M. The mixture was prepared with a magnetic stirrer for 1 h at room temperature (22 °C) under ambient conditions. Thus, aqueous colloidal solutions of CQD1-3+PP hybrid materials were obtained.

### 2.4. Characterization

To obtain images of powdered CQDs, transmission electron microscopy (TEM) was used using the annular dark-field imaging method of sample mapping of scanning electron microscopy (HAADF STEM) on a Tecnai Osiris microscope (Thermo Fisher Scientific, Waltham, MA, USA) at an accelerating voltage of 200 kV.

The elemental composition and chemical and electronic state of the CQDs’ atoms were studied using X-ray photoelectron spectroscopy (XPS) using a Thermo Fisher Scientific Escalab 250Xi (Thermo Fisher Scientific, Waltham, MA, USA).

The hydrodynamic sizes and zeta potentials of the studied samples in water were investigated using a Malvern ZetaSizer Nano ZS device (Malvern, Worcestershire, UK) equipped with a laser with a wavelength of 633 nm; the signal was recorded at an angle of 173°.

The IR absorption spectra were recorded on a Bruker Invenio R FTIR spectrometer (Bruker, Mannheim, Germany) equipped with a diamond Attenuated Total Reflectance (ATR) attachment in the range from 500 to 4500 cm^−1^ with a resolution of 2 cm^−1^. The experimental setup was as follows: a drop (2 μL) of an aqueous solution of CQD or CQD+PP was placed on a diamond crystal of the FTIR spectrometer ATR attachment. Then, for 10 min, the drop was dried in a constant air flow, and the IR spectrum of the resulting powder was measured.

The absorption spectra were measured using a Shimadzu UV-3600 spectrophotometer (Shimadzu, Kyoto, Japan) in the wavelength range of 200–500 nm with a resolution of 1 nm (scanning speed: medium). A quartz cuvette with deionized water was placed in the reference channel.

The photoluminescence spectra were recorded using a Shimadzu RF-6000 spectrofluorometer (Shimadzu, Kyoto, Japan), with excitation wavelengths ranging from 250 to 450 nm in 5 nm increments. The registration of the PL emission spectra was carried out in the wavelength range from 300 to 700 nm with a step of 1 nm, with a scanning speed of 6000 nm/min at low sensitivity. The luminescence quantum yield values were determined using the standard dye method [26]. Quinine sulfate in an aqueous solution of sulfuric acid with a concentration of 0.05 M was chosen as the standard dye (the value of the PL quantum yield upon excitation with radiation with a wavelength of 350 nm is 58% [26]).

The steady-state photoluminescence spectra of hybrids were measured using a modular spectrofluorometer Fluorolog-3 (Horiba, Osaka, Japan) with a continuous-wave Xe lamp as an excitation source. Luminescence kinetics measurements were performed using light emission diode LED340 (wavelength 340 nm, pulse width 1.2 ns, repetition rate 1 MHz). Lifetimes were calculated using the TCSPC technique.

^1^H, ^13^C, ^31^P NMR spectra were recorded in CD_3_OD on a Bruker 500 MHz Avance spectrometer (Bruker, Mannheim, Germany) at frequencies 499.91 (^1^H), 125.70 MHz (^13^C), 202.37 MHz (^31^P). The final concentration of product for NMR analysis was 20 mg in 0.55 mL deuterated solvent.

### 2.5. BChE Activity Measurements

Biological activity was measured using the EasyCheck-Micro bioanalytical platform for monitoring neurotoxic compounds (Kronas, Russia) on planar thiol-sensitive sensors modified with MnO_2_, according to the method [27,28]. The method is based on the interaction of the butyrylcholinesterase (BChE) enzyme and the butyrylthiocholine (BTC) substrate (Scheme S1).

To determine the inhibition of the studied compounds, working solutions of the butyrylcholinesterase enzyme and butyrylthiocholine were prepared in advance. Before each inhibition measurement, preliminary measurements of BChE activity were performed to normalize the enzyme activity. All measurements were performed three times under the same conditions.

The enzyme was incubated with the potential inhibitor for 10 min, then 10 µL of the substrate solution was added to the mixture. Incubation with the substrate was also carried out for 10 min (during this interval, the enzymatic hydrolysis reaction proceeds linearly). After 10 min, the electrode was transferred to the test tube, and the current value characterizing butyrylcholinesterase (BChE) activity after interaction with the inhibitor (I, nA) was recorded.

Stock solutions of BChE (1 mg/mL) and the substrate, butyrylthiocholine chloride (0.5 M), were prepared prior to the assay. To evaluate inhibitory potential, aliquots of the test inhibitor (PP) were added to a microtube containing HEPES buffer (pH 7.5) supplemented with bovine serum albumin (BSA, 1 mg/mL). The enzymatic reaction was initiated by the addition of the substrate and carried out for 10 min; this interval was selected to ensure the linearity of the hydrolysis reaction. Control measurements (blanks) were performed without the inhibitor to normalize enzyme activity for each trial. According to literature data, the relative error of the IPC-micro neurotoxin analysis does not exceed 10%.

### 2.6. Laser Irradiation of the Samples

Laser irradiation experiments were conducted using a Coherent MBD-266 solid-state laser (Santa Clara, CA, USA) operating in continuous-wave mode (λ = 266 nm, output power 30 mW). The beam was defocused to a diameter of 10 mm and directed through a quartz cuvette with a 1 cm optical path length containing the sample solutions. All irradiations were performed for a fixed duration of 60 min under identical conditions.

## 3. Results

### 3.1. Characterization of PP

Our investigations have demonstrated that a diverse range of phosphonates can be classified as photoswitchable compounds, as they exhibit conformational transitions upon photoirradiation through phosphonate group rotation typical for phosphorylated arylamynomalonates [20] and thiazolotriazol [19]; or cis-trans isomerization (for O-functionalized vinyl phosphonates) [21]. Furthermore, these phosphonate compounds were found to possess intrinsic bioactivity towards BChE inhibition, with the magnitude of this bioactivity being directly determined by the conformational state of the molecule. These findings significantly expand the traditional systematization of photoswitches beyond well-known families such as azobenzenes, diarilethenes, stilbenes [29], and introduce phosphonates as a promising new class of conformationally addressable scaffolds with additional bioactive functions.

**Figure 1:**
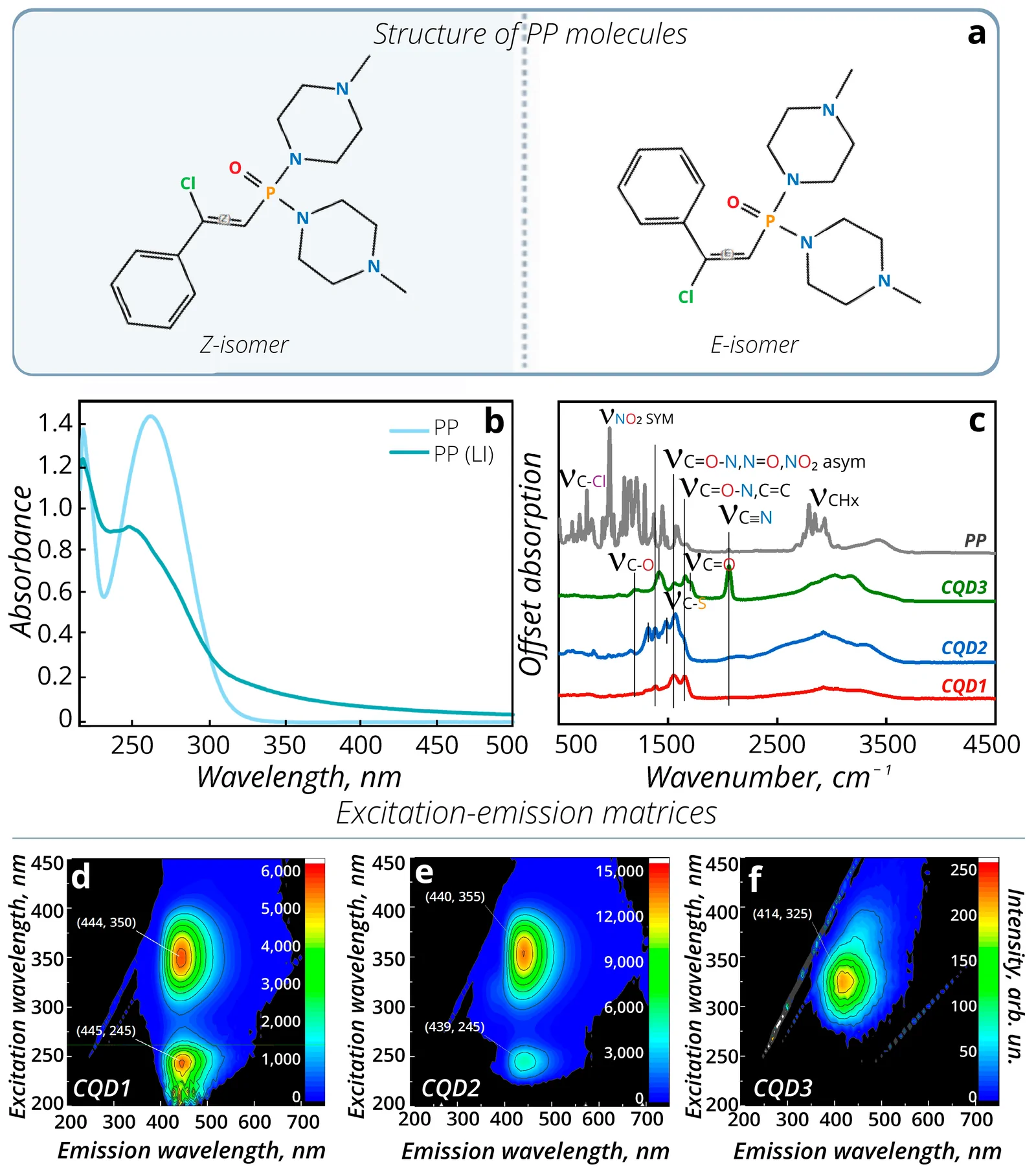
(**a**) Structure of PP molecules before (*Z*-isomer) and after laser irradiation (*E*-isomer) in accordance with NMR, (**b**) UV-VIS absorption spectra of the PP water solution, (**c**) IR absorption spectra of the initial components of CQD+PP hybrids evaporated from aqueous solutions, (**d**–**f**) excitation-emission matrices of CQDs.

Considering the ‘vinyl phosphonate’ moiety as a functional scaffold providing photoswitchable functions, we synthesized an N-functionalized vinyl phosphonate with an amine radical, such as 1-methylpiperazine. 1-methylpiperazine is a basic intermediate for the synthesis of a wide range of active pharmaceutical ingredients. The phosphonate derivative that is symmetrical (2-chloro-2 phenylethenyl)(diamine)phosphine oxide (PP) was synthesized by reacting 2-phenylethynylphosphonic acid dichloride with the corresponding amine in the presence of trimethylamine in accordance with Scheme S2. Mass and IR spectroscopy confirmed formation of the target PP compound (Supporting Information). Structural characterization with NMR homo- and heteronuclear analysis showed that the as-synthesized PP is the *Z*-isomer with structure presented in Figure 1a. The ^31^P NMR spectrum of PP in solution shows a single signal at δ_P_ +20.84 ppm, indicating the single *Z*-isomeric form (see Supporting Information).

Since the photoswitchable motif is a structural feature of the PP compound, tests were carried out to investigate its photoswitching capability. According to the absorption spectra shown in Figure 1b, the absorption band of PP is centered at 260 nm. Irradiation of an aqueous solution of PP with resonant laser light at 266 nm for 60 min revealed pronounced changes in the absorption spectrum, manifested by a decrease in intensity and a blue shift of the absorption band (Figure 1b). The absorption spectrum of PP-LI likely comprises contributions from both *Z*- and *E*-isomers. The experimental spectra indicate that the E-isomer differs markedly from the initial *Z*-isomer, exhibiting a lower absorbance and an absorption peak centered at a shorter wavelength. The ^13^C NMR characterization of the laser-irradiated PP compound showed distinct differences in signals in the downfield region (Table 1). Specifically, a quadruple phosphorus coupling constant is present for the ortho-carbon of the phenyl ring, a feature absent in the original non-irradiated compound. In the proton spectrum of the laser-irradiated PP compound, the signal corresponding to the second phosphorus-coupling constant of the vinyl proton is shifted upfield, while the value of the coupling constant itself remains largely unchanged. The observed changes in ^13^C NMR and phosphorus spectra testify to new isomer formation.

Given that the structure of the PP under investigation contains an unsaturated bond, suggesting the possibility of light-induced isomeric transitions, we examined the structure of both irradiated and non-irradiated samples using two-dimensional ^1^H–^1^H NOESY NMR spectroscopy (Figure S1). Accordingly, the ^1^H–^1^H NOESY spectrum of the *Z*-isomer reveals a nuclear Overhauser effect between the ortho-proton of the aromatic ring and the =CH fragment, while no such effect was detected for the *E*-isomer. Thus, ^1^H, ^31^P, and ^13^C NMR data confirm the formation of the *E*-isomer upon laser irradiation of PP (Figure 1a). The integrity of the molecular framework upon irradiation is confirmed by the preservation of all parent signals in ^1^H, ^13^C, and ^31^P NMR spectra, with the latter showing only one new signal corresponding to the isomeric form. No extraneous signals indicative of decomposition products were detected, ruling out photodegradation under our experimental conditions. IR spectroscopy further supports the *Z*-*E* conformational transition (Figure S2 and Table S1).

As recent studies have demonstrated the considerable potential of photoswitchable molecules and their extended-functionality hybrids, further interest lies in endowing PP with a luminescence function, specifically by creating a hybrid comprising a luminescent core and a photoswitchable PP shell. Importantly, the nature of the bonding between the photoswitch and the core critically determines the resulting functionality and shapes fundamental insights into photoswitching ability under sterically constrained conditions. An important feature of carbon quantum dots is their stable luminescence and the ability to finely tune their properties through variation in functional groups. The latter aspect is of particular interest in the design of photoswitchable hybrids based on carbon quantum dots.

### 3.2. Characterization of CQDs

To provide carbon quantum dots with functional group variants, CQD samples were synthesized by the hydrothermal method from different precursors at different reaction times and temperatures. Figure S3 shows images of powdered CQD1, CQD2, and CQD3 obtained using an electron microscope. As can be seen from the microscopy results, the samples of CQDs synthesized from CA and EDA demonstrate the presence of quasi-spherical particles characterized by sizes of ~5 nm with an interplanar distance of about 0.26 nm, which agrees with the (100) plane lattice of graphitic carbon [30]. In the sample from CA and TU (CQD3), the found particles are characterized, on average, by a smaller size of ~3 nm. Zeta potentials and hydrodynamic sizes of CQDs in water showed monodisperse distributions; their average values are presented in Table 2. The observed discrepancy between the ~1 nm hydrodynamic sizes from DLS and the larger particle sizes from HRTEM images, where only rare large crystalline particles are resolvable due to technical limitations, suggests that most CQDs are ~1 nm in size.

According to the XPS results (Figure S4a, Table 2), the synthesized CQD samples consist mainly of carbon; the content of nitrogen and oxygen atoms is quite similar, and each is about 20% of the total atom amount. It was found that the CQD3 sample doped with sulfur atoms contains about 6% of them. The decompositions of the C, N, and O 1s spectra into Voigt profile components (convolutions of Gaussian and Lorentzian functions) were performed by the Levenberg–Marquardt method [31]. The decomposition of individual peaks corresponding to different atoms is shown in Figure S4b–d.

CQDs functional groups were characterized with IR absorption spectroscopy (Figure 1c; the detailed description is presented in SI). In all CQDs’ spectra, bands of vibrations of amide bonds (C=O)-N are observed, which are the result of the formation of a peptide bond between the carboxyl groups -COOH of CA and the amino groups -NH_2_ of EDA or TU at the initial stage of synthesis. CQD1 has predominantly (C=O)-N, -N=O, and -CH_x_ groups on the surface with a small number of -NO_2_ and -C≡N, whereas CQD2 is characterized by predominantly (C=O)-N, -NO_2_, -N=O, and -CH_x_ groups. CQD3, doped with sulfur in addition to nitrogen, has a large number of -C≡N groups (probably in the thiocyanate group -S-C≡N), C-S bonds, (C=O)-N, and a small number of -N=O groups in its structure. The main functional groups of CQD1-3 are summarized in Table 2.

In accordance with absorption spectroscopy (Figure S5), CQD1 and CQD2 are characterized by broad absorption peaks in the region of 250 and 343 nm, corresponding to the π–π* transition of sp^2^ domains and the n–π* transition of multi-conjugate C=O of the CQDs, respectively [32]. The absorption spectrum of CQD3 shows an absorption band in the region of 220 nm, which can be attributed to a specific molecular π-π* transition of aromatic sp^2^ conjugated domains [33]. PL excitation-emission matrices of CQDs (Figure 1d–f) reveal broad photoluminescence bands with maxima in the region of 445 nm upon excitation by radiation with wavelengths of 245 and 350 nm for both CQD1 and CQD2. While CQD3, doped with both nitrogen and sulfur atoms, has structureless photoluminescence with a maximum in the region of 414 nm upon excitation with a wavelength of 325 nm. QY of CQDs’ luminescence at excitation wavelengths of 350 nm for CQD1 and CQD2, and 325 nm for CQD3 were 98%, 71%, and 12%, respectively (Table 2).

Thus, comprehensive characterization of the synthesized CQDs revealed that all obtained samples exhibit the essential functional property—luminescence—while differing in their surface functional groups, which will enable studies on how the nature of interactions can be modified during the formation of the CQD+PP hybrid.

### 3.3. CQD+PP Characterization

**Figure 2:**
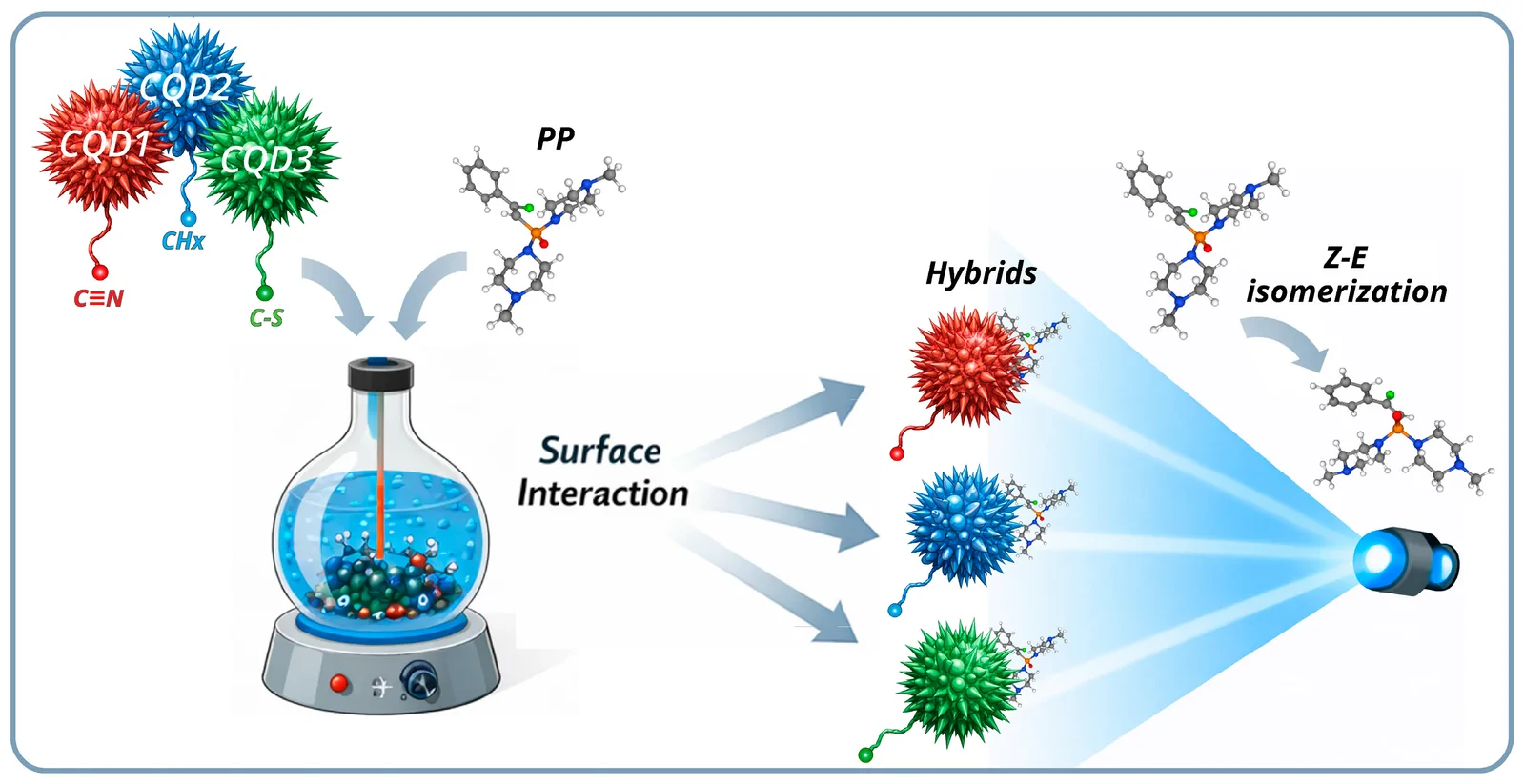
Synthesis of hybrid CQD+PP nanostructures.

To integrate intrinsic photoswitchable functions of PP compounds conjugated with luminescence of CQD, their hybrids were synthesized by mixing an aqueous colloidal suspension of each CQD with 1,1′-([(Z)-2-chloro-2-phenylethinyl]phosphoryl)dimethylpiperazine at ambient conditions for 60 min (Figure 2). Then, the obtained samples were studied with absorption, luminescence, and FTIR spectroscopies, as well as IPC-micro neurotoxin amperometric analysis.

#### 3.3.1. UV-VIS Absorption

**Figure 3:**
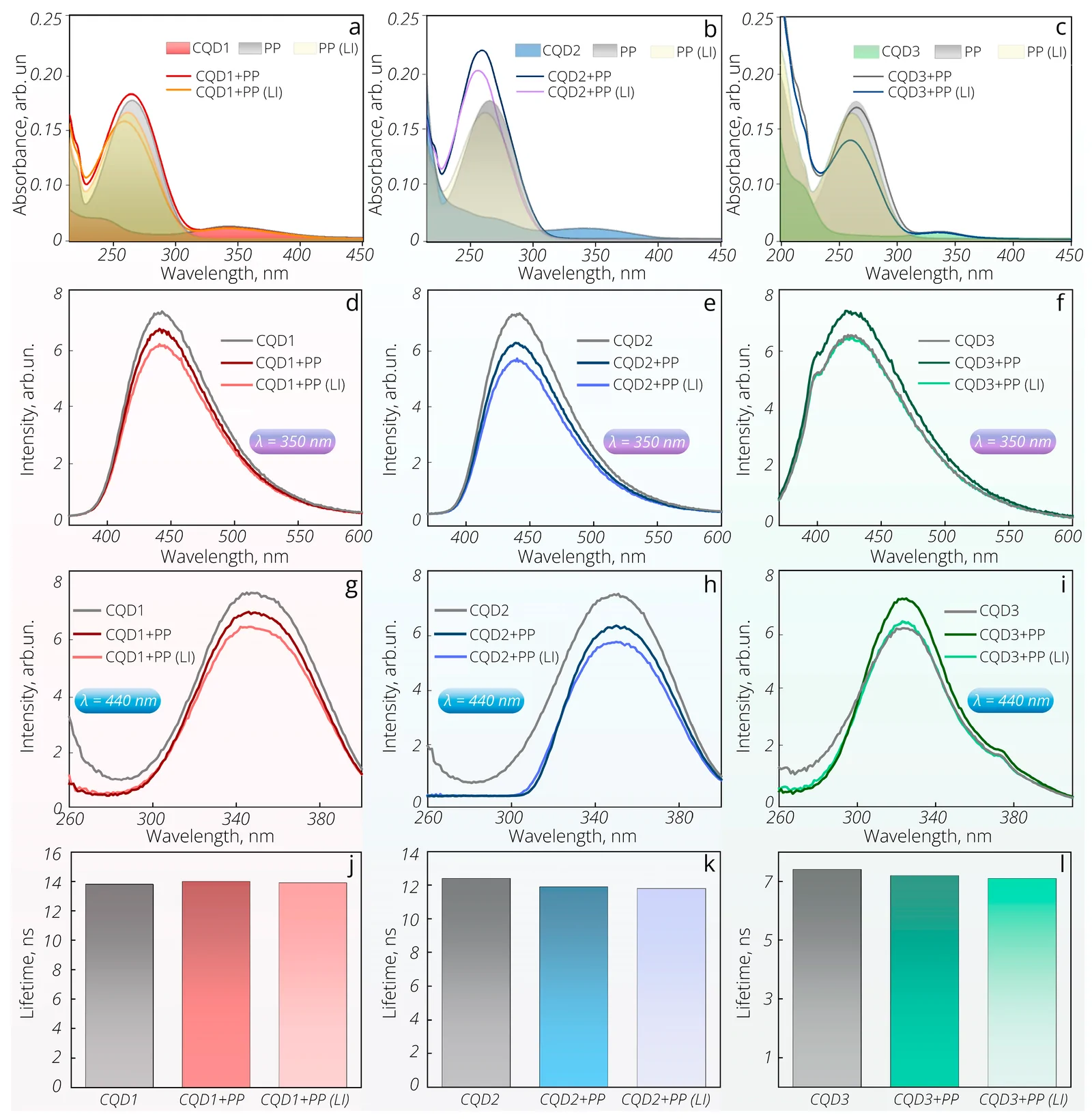
The absorption spectra of the hybrid materials and their individual components before and after laser irradiation for (**a**) CQD1, (**b**) CQD2, (**c**) CQD3. (**d**–**f**) Emission, (**g**–**i**) excitation spectra, and (**j**–**l**) lifetimes of hybrids before and after laser irradiation. A shoulder in the short-wavelength region (~399 nm) in Figure 3f is ascribed to a Raman peak from the aqueous solvent.

Figure 3a–c presents the absorption spectra of individual components (PP and CQD1-3) and their hybrids before and after laser irradiation. The spectra of the hybrids are characterized by an intense absorption band centered at 260 nm and a lower-intensity band at 340 nm, attributed to the PP and carbon quantum dot components, respectively. The observed variation in PP-originated peak intensity and position for hybrids in comparison with pristine PP testifies to the interaction between the components PP and CQD and confirms hybrid formation. It should be noted that the same feature is observed for laser-irradiated CQD+PP hybrids in comparison with laser-irradiated PP, thus testifying to the preservation of hybrid integrity after laser treatment.

Laser irradiation for 60 min induces a similar decrease in absorption for all hybrids, accompanied by a slight blue shift of the phosphonate band to 256 nm. This effect mirrors the spectral changes observed upon irradiating the pure phosphonate aqueous solution. In contrast, the absorption band corresponding to the CQDs remains unaltered, indicating no detectable photoeffect on these nanoparticles.

#### 3.3.2. Photoluminescence Properties

Luminescence spectroscopy demonstrated overall preservation of the CQD luminescent properties as components of the CQD+PP hybrids (Figure 3d–f). However, a reduction in luminescence intensity was observed for CQD1+PP and CQD2+PP, and a slight enhancement for CQD3+PP in comparison with CQD1-3. The observed phenomenon is likely attributed to the effect of PP on the local environment of the CQD luminescent center due to hybrid formation. The difference in luminescence behavior—decrease for CQD1-2+PP and increase for CQD3+PP—may indicate distinct mechanisms of hybrid formation.

Laser irradiation of all hybrid samples results in a measurable decrease in emission intensity, with the observed reduction ranging from approximately 10% to 15%. Notably, the spectral position of the broad emission band corresponding to the CQDs remains unaffected by either hybrid formation or subsequent laser irradiation. Excitation spectra reveal distinct maxima for the hybrids: a band centered at 350 nm for CQD1+PP and CQD2+PP, and one at 325 nm for CQD3+PP (Figure 3g–i). In all cases, the excitation maximum is unaffected by subsequent laser irradiation.

The luminescence decay profiles of CQD1-3 and their corresponding hybrid samples, both before and after laser irradiation, were recorded using time-correlated single photon counting (TCSPC). An LED340 source (λ_ex_ = 340 nm, pulse duration 1.2 ns) was used for excitation. The experimental data were fitted with a single-exponential function for CQD1 and CQD2, and a biexponential function for CQD3. For biexponential fitting, the average lifetime was calculated for comparison using the formula:$\tau_{av}=\frac{I_{1}\tau_{1}^{2}+I_{2}\tau_{2}^{2}}{I_{1}\tau_{1}+I_{2}\tau_{2}}$, where *I*_1_ and *I*_2_ are the pre-exponential factors; τ_1_ and τ_2_ are the corresponding lifetimes [26]. The resulting lifetimes were 13.8 ns (CQD1), 12.5 ns (CQD2), and 7.3 ns (CQD3) (Figure 3j–l). Notably, hybrid formation and subsequent laser irradiation had only a minor impact on the lifetime. The most significant change was observed for CQD2+PP (LI), where the lifetime decreased to 11.7 ns.

The results of this study clearly demonstrate that the luminescence properties of the CQDs are largely retained for the CQD+PP hybrids. Exposure to 266 nm laser irradiation, which drives photoisomerization of the PP component, leads to only a modest reduction in CQD emission intensity (10–15%), while the spectral position of the broad emission band, the excitation maxima, and the excited-state lifetimes remain virtually unchanged. This pronounced photostability establishes the CQDs as reliable optical probes that can be monitored irrespective of whether the hybrid has been irradiated. Moreover, the clear separation between the CQD excitation band (350 nm) and the PP photoswitching wavelength (266 nm) permits independent photoisomerization of the PP compound and excitation of CQD luminescence. Consequently, the luminescence of the hybrids can be read out without perturbing the structure of the PP component, enabling non-invasive monitoring and orthogonal control of photoswitching and emission.

Thus, optical characterization of the CQD+PP hybrids provided critical insights into both the formation of the hybrid structure and the mutual electronic influence between the components. Spectroscopic analyses revealed a clear interplay between the components, manifested as changes in the absorption and emission features relative to the pristine materials, providing indirect evidence of hybrid formation. Importantly, despite this evident component interaction, the characterization distinctly demonstrated the retention of the individual functional properties essential for practical application. Specifically, the PP molecules preserved their reversible photoswitching capability upon optical stimulation, while the CQD carrier maintained its intrinsic luminescence. Moreover, absorption and luminescence spectra revealed peculiarities of CQD1-3+PP hybrids in dependence on CQD functional groups. To uncover the impact of functional groups on hybrids’ individual differences, further characterization with FTIR spectroscopy was carried out.

### 3.4. CQD+PP Hybrids Formation

To clarify the mechanisms of CQD+PP hybrid formation, the FTIR absorption spectra of CQD+PP and the spectra of individual components were studied, along with the spectra of hybrids from which the PP spectrum was subtracted with a certain factor, selected manually to minimize discrepancy with CQD spectra from reasonable considerations (Figure S6 and description).

For CQD1+PP and CQD2+PP hybrids, the IR absorption spectra of CQDs are virtually identical to those of CQD+PP hybrids after subtraction of the PP spectrum (Figure S6a,b). The only discernible deviation that cannot be accounted for by this subtraction occurs around 960 cm^−1^, where a band associated with ν(PN) stretching vibrations and nearby ν(C-N-C) modes in the PP ring shifts to a higher frequency. This shift is attributed to a subtle bending or rotation of the PP rings relative to one another. Coupled with the absence of any other changes in the absorption bands of either component, this observation indicates that physical adsorption is the primary mechanism underlying the formation of hybrids between PP and both CQD1 and CQD2.

In contrast, the CQD3+PP hybrids exhibit markedly different FTIR spectra compared to those of the individual precursors (Figure S6c). Substantial spectral changes are observed not only in the fingerprint region (500–1500 cm^−1^) but also in the multiple-bond (1500–2500 cm^−1^) and X-H stretching (2500–4000 cm^−1^) regions. Specifically, the intensity of the ν(CHx) absorption bands between 2650 and 3000 cm^−1^ decreases significantly, accompanied by the appearance of a broad shoulder spanning 2300–2700 cm^−1^. This feature likely arises from strongly hydrogen-bonded carboxyl groups (–(C=O)–O–H···X), stretching vibrations of N–H in quaternary ammonium salts, or stretching modes of NH_2_^+^ and NH^+^ species [34]. Additionally, a minor peak near 2650 cm^−1^ is consistent with the stretching vibrations of P–OH in hydrogen-bonded phosphoric esters. The FTIR spectra of the CQD3+PP hybrid are so extensively transformed that the analysis is most reliably guided by the few remaining invariant features. Specifically, the hybrid retains a band at 1448 cm^−1^, assigned to δ(HCH) deformation vibrations of the N-ring (the non-aromatic ring of PP), along with a doublet near 1570 cm^−1^ arising from ν(C=C) alkene vibrations coupled to ν(C=C) modes in the benzene ring. The displacement or complete loss of all other bands points to a substantial structural reorganization. Collectively, these spectral changes provide strong evidence that the CQD3+PP hybrid forms via robust chemical interactions involving new chemical bond formation.

### 3.5. Transformation of CQD+PP Hybrids as a Result of Laser Irradiation

**Figure 4:**
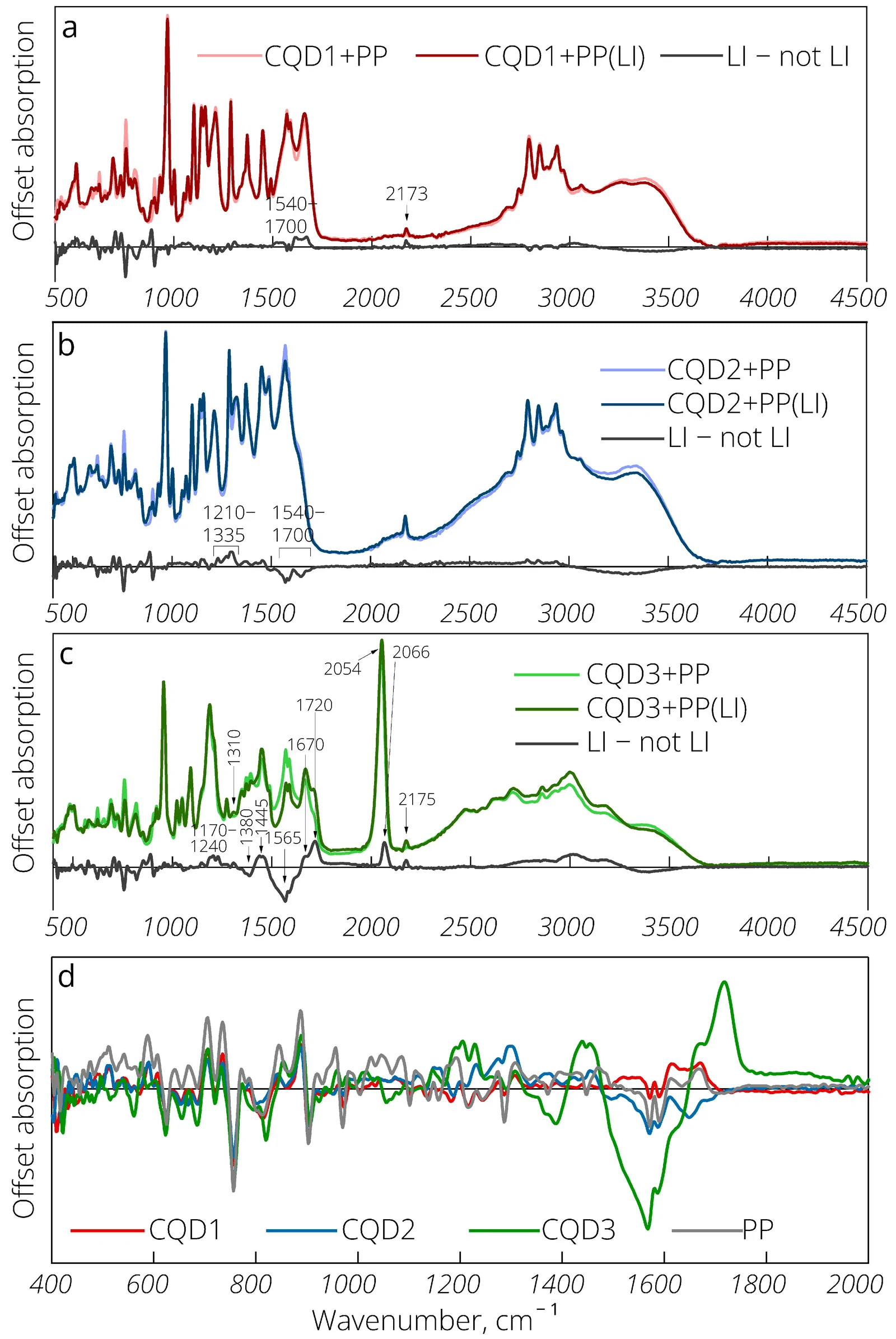
(**a**–**c**) FTIR spectra of CQD+PP hybrids before and after laser irradiation (LI) and their difference spectra, LI spectra being adjusted to minimize the divergence, (**d**) overlay of the resulting difference spectra.

A comparative analysis of the FTIR absorption spectra of CQD+PP hybrids before and after laser irradiation was performed using difference spectra. Figure 4a–c shows the spectra of the hybrids before and after irradiation, as well as the difference spectrum between the laser-irradiated and initial ones. The overlay of all the difference IR spectra is presented in Figure 4d. It is obvious that positive values of the difference curves correspond to an increase in absorption in the corresponding region as a result of irradiation, and vice versa.

Figure 4 shows that the difference spectra of all hybrids in the range up to 1000 cm^−1^ largely replicate each other, as well as the similar difference spectrum of PP. For CQD1+PP and CQD2+PP hybrids, they are almost identical. In this region, the distinct maxima correspond exclusively to the absorption of PP, and the observed changes are associated with its transformation under laser irradiation. Moreover, the small magnitude of these changes and the alternation of maxima and minima indicate a band shift rather than the appearance or disappearance of new vibrations. Thus, the FTIR spectroscopy data indicate that irradiation most likely leads to conformational changes in PP rather than structural ones.

In the frequency range above 1000 cm^−1^, the difference spectra of the hybrids reveal features that depend on the type of CQD used. Specifically, for the CQD1+PP hybrid after irradiation, notable changes are confined to the 1540–1700 cm^−1^ region. A negative feature, consisting of two peaks below 1600 cm^−1^, arises from modifications in the PP bands—specifically, ν(C=C) of the alkene moiety coupled with ν(C=C) vibrations of the benzene ring. The intensity increase between 1600 and 1700 cm^−1^ reflects a strengthening of ν(C=C) modes. These vibrations likely originate from similar C=C bonds located in distinct local environments, and laser irradiation induces a redistribution between these environments.

The difference spectrum of the CQD2+PP hybrid exhibits changes similar to those observed for CQD1+PP (Figure 4a,b,d). In contrast to the latter, however, the CQD2+PP spectrum shows a decrease in the intensity of ν(C=C) vibrations in the 1540–1700 cm^−1^ region rather than an increase. Concurrently, an increase in band intensity is observed in the 1210–1335 cm^−1^ region, associated with v(=C-C_Ph_), δ(CCH), τ(HCH), δ(NCH), δ(CCH), v(C-N-C), and v(C-O-C).

The most pronounced changes are observed in the spectra of the CQD3+PP hybrid (Figure 4c,d). After irradiation, a weak peak appears near 2175 cm^−1^, assigned to C≡N bonds, although not to -S-C≡N (notably, this feature is also present in the spectra of other CQD hybrids after irradiation). The peak at 2054 cm^−1^, attributed to -S-C≡N, develops a shoulder with a maximum at 2066 cm^−1^, while the intensity of the original peak remains unchanged. These features may reflect vibrations of the same S-C≡N groups adopting a new configuration. The band at 1720 cm^−1^, arising from C=O stretching vibrations in a C-(C=O)-C environment, intensifies and/or becomes more resolved. Concurrently, a significant decrease in absorption intensity is observed over the 1480–1650 cm^−1^ range, with a maximum near 1565 cm^−1^, primarily attributable to the disappearance of amide group vibrations -(C=O)-N. Considering these differences in spectral changes in the 1720 cm^−1^ region, it can be suggested that laser irradiation of CQD3+PP hybrid alters the local environment of the C=O group, specifically by replacing N with C, which can also be considered as a result of PP conformational change. PP photoisomerization within CQD3+PP takes place with the switching of chemical bonds.

Summing up the infrared spectroscopy results, it can be concluded that for CQD1 and CQD2 bearing CH_x_, -NO_2_, -N=O functional groups on their surface, hybrid formation occurs via physical sorption. Notably, the laser irradiation process applied to the CQD1+PP and CQD2+PP hybrids does not lead to their degradation. Moreover, the observed minor changes in the FTIR spectra point to conformational rearrangements of the PP molecule rather than any chemical decomposition, indicating a degree of structural flexibility under optical stimulation.

In contrast, for CQD3 with C≡N, S-C≡N, COOH, C-S, C=O functional groups, hybrid formation proceeds through chemical bonding. Despite this covalent integration, the ability of PP to undergo photoisomerization upon irradiation is retained. Thus, while the mode of interfacial interaction differs fundamentally across the series—physical sorption for CQDs1-2 versus chemical grafting for CQD3—the functional integrity of the photoswitchable PP is preserved in all cases.

### 3.6. BChE Inhibition

**Figure 5:**
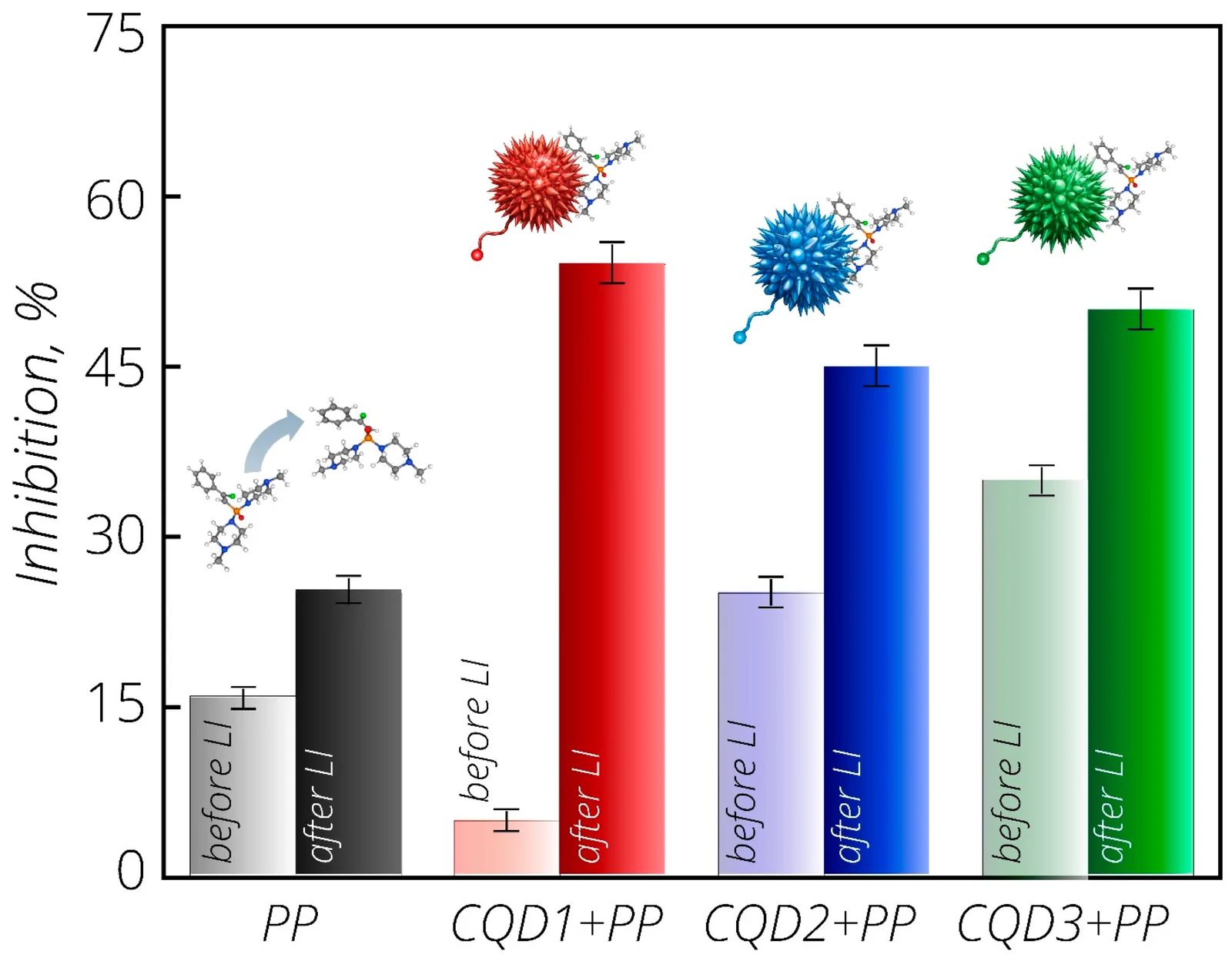
BChE inhibition value for PP and CQD+PP hybrids before and after laser irradiation.

Since the inhibition of butyrylcholinesterase represents a fundamental functional attribute of the pristine PP molecules, the preservation of this bioactivity upon hybrid formation—as well as its modulation after laser irradiation is a key practical issue for potential applications. Thus, the BChE inhibition of three types of CQD1-3+PP hybrids as well as their components (PP, CQD1-3) before and after laser irradiation was studied (Figure 5). It was found that pristine components that are CQD1-3 alone did not exhibit BChE inhibition either before or after irradiation. In contrast, PP at a concentration of 10^−4^ M demonstrated 15% inhibition and a twofold increase after laser irradiation (PP-LI) (15–30%).

In spite of the observed zero bioactivity of pristine CQDs 1–3, investigation of BChE inhibition for CQD+PP hybrids revealed a pronounced modulatory effect imparted by the carbon quantum dots. Specifically, the BChE inhibition of all hybrids was distinctly different from that of pristine PP alone. Inhibition values varied significantly depending on the hybrid type, with CQD1+PP exhibiting the lowest inhibition at approximately 5%, followed by a gradual increase for CQD2+PP and CQD3+PP hybrids, which reached 28% and 32%, respectively. Furthermore, exposure of the hybrids to laser irradiation revealed the preservation of a systematic increase in bioactivity typical of pristine PP. At the same time, a substantial influence of the luminescent carbon dot carrier on the magnitude of irradiation-induced changes in hybrids’ bioactivity was again established. In particular, a more than 10-fold increase was observed for CQD1+PP (from 5% to 55%), along with approximately 2-fold and 1.5-fold enhancements for CQD2+PP and CQD3+PP hybrids, respectively.

Finally, the overall experimental findings allow us to conclude that the functional properties of both components—the luminescent nanocarrier and the photoswitchable bioactive molecule—are preserved upon their integration into the hybrid system. Furthermore, the observed mutual influence of the components, manifested in variations in optical properties and bioactivity values for the hybrids, indicates the formation of stable structures that remain intact after optical stimulation. The most notable observation concerns the pronounced effect of the quantum dots’ surface functional groups on the hybrid formation mechanism and on the magnitude of the bioactivity window before and after photoswitching. Interestingly, in both physisorption and chemisorption modes, the PP molecule retains its ability to undergo photoswitching; however, likely due to steric hindrance in the case of the CQD3+PP hybrid involving chemisorption, a narrower bioactivity window is observed. In contrast, physical binding of the hybrid components provides a larger dynamic range of bioactivity variation, with the highest from 5% to 55% for CQD1+PP. These results underscore the high potency of the observed phenomenon: by strategically selecting either chemisorption or physisorption as the mode of interaction between organic photoswitches and carbon quantum dots, one can achieve fine control over the dynamic range of bioactivity modulation. This capability opens a versatile route toward creating photoswitchable hybrid materials with tailored on/off ratios for diverse therapeutic applications.

## 4. Conclusions

In summary, we demonstrated the synthesis and comprehensive characterization of aqueous colloidal solutions of organic-inorganic hybrids composed of carbon quantum dots and a phosphonate compound. A series of CQD samples were synthesized by the hydrothermal method from different precursors under various synthesis conditions. FTIR spectroscopy clearly demonstrated differences in the functional groups inherent in different CQDs. Moreover, optical properties of CQDs significantly differed—absorption and emission bands notably shifted, and photoluminescence quantum yield dramatically dropped in the row CQD1-CQD2-CQD3. The phosphonate compound exhibited *Z*-*E* isomerization upon laser irradiation and pronounced biological activity towards BChE.

CQD+PP hybrids were synthesized by simply stirring the components under ambient conditions. A detailed analysis of the IR spectra indicates that physical adsorption is the predominant mechanism governing the formation of the CQD1+PP and CQD2+PP hybrids. In contrast, the substantial spectral changes observed for the CQD3+PP hybrid provide strong evidence for the formation of new chemical bonds. Luminescence studies confirmed that the steady-state and kinetic emission characteristics of the hybrids closely resemble those of the corresponding initial CQDs. Laser irradiation of all hybrid samples led to a measurable decrease in emission intensity, with the reduction ranging from approximately 10% to 15%. FTIR spectroscopy further revealed that laser irradiation of the CQD1+PP and CQD2+PP hybrids primarily induces conformational changes in the phosphonate, rather than structural alterations, whereas irradiation of CQD3+PP causes significant modifications in the vibrational bands associated with C=C, C=O, and C–H bonds. All hybrids retained biological activity, with the specific type of CQD governing the mode of inhibition: the CQD2+PP and CQD3+PP hybrids enhanced bioactivity compared to the pristine phosphonate, while CQD1+PP exhibited reduced activity. Laser irradiation led to a remarkable increase in BChE inhibition for all hybrids, with fold enhancements of 10 (CQD1+PP), 2 (CQD2+PP), and 1.5 (CQD3+PP). It can be concluded that the type of binding mode within the CQD+PP hybrid significantly modulates the range of photomodulated bioactivity. This finding demonstrates the high potency of the proposed hybrid platform, revealing that simple variation in the component-binding mode enables precise, on-demand tuning of the bioactivity window—a critical advantage for designing next-generation photopharmacological agents.

## Figures and Tables

**Table 1:** Basic phosphorus constants of compound PP before and after laser irradiation in the ^13^C NMR and ^1^H NMR spectra.

	*Z*-Isomer		*E*-Isomer	
	*J*_CP_	ppm	*J*_CP_	ppm
P-CH=C	^1^*J*_CP_ = 161.74 Hz	114.91 d	^1^*J*_CP_ = 154.04 Hz	117.94 d
P-CH=C	^2^*J*_CP_ = 14.30 Hz	137.25 d	^2^*J*_CP_ = 3.65 Hz	136.86 d
*ipso*-C_Ph_	^3^*J*_CP_ = 1.93 Hz	149.72 d	^3^*J*_CP_ = 14.64 Hz	151.03 d
*o*-C_Ph_	-	128.43 s	^4^*J*_CP_ = 1.10 Hz	128.84 d
	***J*** **_HP_**	**ppm**	***J*** **_HP_**	**ppm**
P-CH	^2^*J*_HP_ = 10.61 Hz	6.58 d	^2^*J*_HP_ = 10.68 Hz	6.40 d

**Table 2:** Physicochemical parameters of CQDs.

Sample	Synthesis Parameters	Hydrodynamic Size, nm	HRTEM Size, nm	Zeta Potentials: Average ± HWHM, mV	Groups and Bonds from FTIR: Many/ Few/ Possible	Atomic Composition (XPS Data)	QY *
CQD1	EDA:CA 2:1, 3 h, 200 °C	1.1 ± 0.2	5.1±0.4	−7.8 ± 3.3	(C=O)-N, -N=O, -CH_x_/ -NO_2_, -C≡N/ -OH, -C=C-	C (60%), N (20%), O (20%)	98%
CQD2	EDA:CA 20:1, 3 h, 140 °C	1.2 ± 0.2	4.9 ± 0.5	−17.0 ± 5.5	(C=O)-N, -NO_2_, -N=O, -CH_x_,/ C-N, N-H/ -OH, -C=C-	C (56%), N (24%), O (20%)	70%
CQD3	TU:CA 1:1, 4 h, 160 °C	1.0 ± 0.2	3.2 ± 0.6	−10.0 ± 4.1	-S-C≡N, (C=O)-N, -COOH/ -N=O, C-O, C=O/ -OH, -C=C-	C (53%), N (17%), O (22%) S (6%)	12%

* The error in QY determination by the used method estimated as 10% of the value.

## Data Availability

Data will be made available on request.

## Supplementary Materials

The following supporting information can be downloaded at: https://www.mdpi.com/article/10.3390/nano16171066/s1. Additional experimental data include synthesis scheme, NMR analyses, UV-VIS absorption, FTIR analysis, HRTEM images, and XPS data. Scheme S1: Enzymatic hydrolysis of butyrylthiocholine; Scheme S2: Synthesis of symmetrical (2-chloro-2 phenylethenyl)(diamine)phosphine oxide (PP); Figure S1: NMR ^1^H-^1^H NOESY spectra of PP after 60 min of 266 nm laser irradiation; Figure S2: IR absorption spectra of PP before and after UV laser irradiation; Table S1: Selected IR absorption bands in the spectra of PP; Figure S3: HRTEM images of CQDs: (a) CQD1, (b) CQD2, (c) CQD3; Figure S4: (a) The XPS survey scans of the synthesized CQDs. (b)-(d) The XPS deconvoluted C1s, N1s, O1s, and S2p peaks of CQDs; Figure S5: Optical density spectra of aqueous solutions of CQD; Figure S6: FTIR spectra of individual CQDs, PP, and their mixtures (as obtained, not normalized, only offset) for (a) CQD1, (b) CQD2, and (c) CQD3.
