# Supplementary material for: Carbon Quantum Dots as a Luminescent Platform for Photoswitchable Bioactive Hybrids: Tuning Butyrylcholinesterase Inhibition Through Functional Group Engineering
Source: Nanomaterials (Basel). 2026 Aug 27;16(17):1066. doi: 10.3390/nano16171066 (PMC13567531; doi:10.3390/nano16171066)
Supplement: Supplementary file 1 [file nanomaterials-16-01066-s001.zip › nanomaterials-4441886-supplementary.pdf]

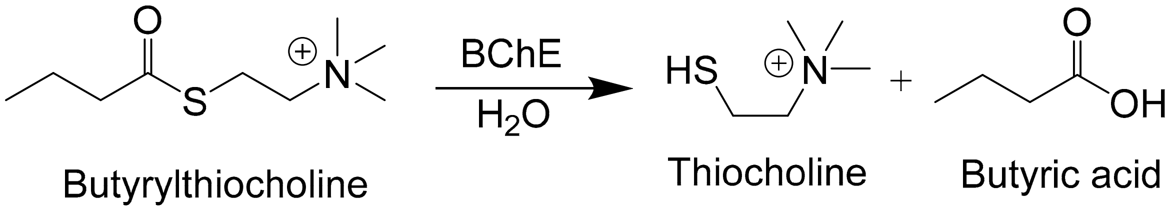

Scheme S1. Enzymatic hydrolysis of butyrylthiocholine.

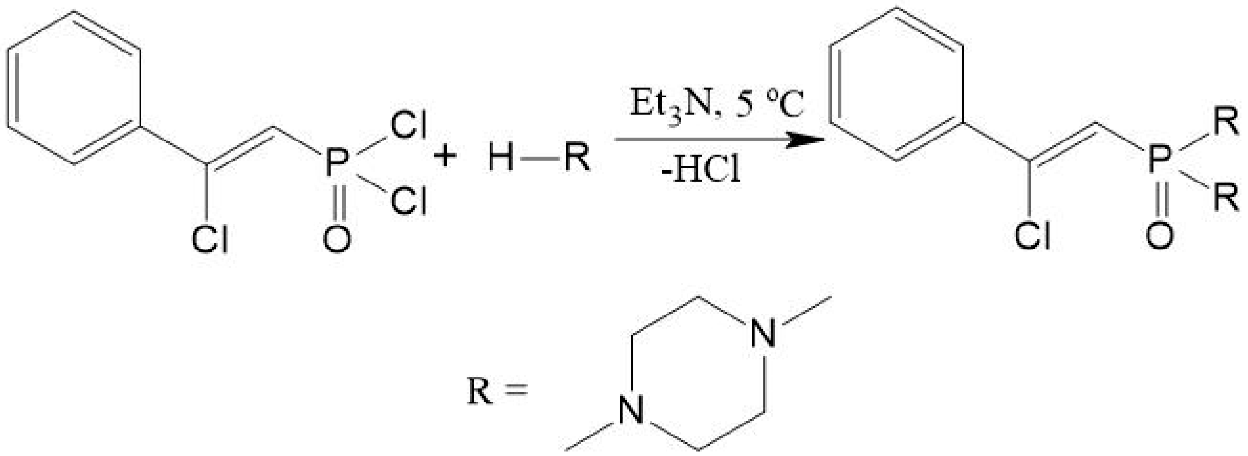

Scheme S2. Synthesis of symmetrical (2-chloro-2 phenylethenyl)(diamine)phosphine oxide (PP)

1,1'-([(*Z*)-2-chloro-2-phenylethinyl]phosphoryl)dimethylpiperazine (PP)

^{1}H NMR spectrum,δ, ppm: 2.33 s (6H, $-CH_3$), 2.49 s (8H, $-CH_2$), 3.24 – 3.23 m (8H, N-CH_2), 6.58 d (1H, =CH-P, $^2J_{HP}$ =10.61 Hz), 7.45 – 7.50 m (3H, *m*-Ph, *p*-Ph), 7.78 dd (2H, *o*-Ph. $^3J_{HH}$= 7.96 Hz, $^4J_{HH}$ = 1.73 Hz).).^{13}C NMR spectrum,δ, ppm: 43.21 ($-CH_3$, $^5J_{CP}$= 2.45 Hz), 44.95 (P-N-CH_2), 54.69 d (CH_2-N, $^3J_{CP}$ = 5.69 Hz), 114.91 d (P-CH, $^1J_{CP}$=161.74 Hz), 126.72 (*m*-C_{Ph}), 128.43 (o-C_{Ph}), 130.58 (*p*-C_{Ph}), 137.25 d (P-CH=C,$^2J_{CP}$=14.30 Hz),149.72 d (*ipso*-$C_{Ph,}$ $^3J_{CP}$ = 1.93 Hz).^{31}P NMR spectrum, δ, ppm: 20.84. Mass-spectrum calculated for $C_{18}H_{28}ClN_4OP$: M 382,1760; found: 383,1762 (M+H)+. FTIR: 685, 727, 759, 780, 813, 907, 918, 952, 970, 1052, 1074, 1101, 1132, 1165, 1185, 1214, 1285, 1332, 1355, 1365, 1445, 1456, 1494, 1570, 1588, 2806, 2846, 2935, 3063 cm^{-1}.

1,1'-([(*E*)-2-chloro-2-phenylethinyl]phosphoryl)dimethylpiperazine (PP-LI)

^{1}H NMR spectrum,δ, ppm: 2.25 s (6H, $-CH_3$), 2.29 – 2.31 m (8H, $-CH_2$), 3.01 – 3.05 m (8H, N-CH_2), 6.40 d (1H, =CH-P, $^2J_{HP}$ =10.68 Hz), 7.42 – 7.47 m (3H, *m*-Ph, *p*-Ph), 7.66 dd (2H, *o*-Ph. $^3J_{HH}$= 7.89 Hz, $^4J_{HH}$ = 1.65 Hz).^{13}C NMR spectrum,δ, ppm: 43.07 ($-CH_3$, $^5J_{CP}$= 2.29 Hz), 44.85 (P-N-CH_2), 54.47 d (CH_2-N, $^3J_{CP}$ = 5.89 Hz), 117.94 d (P-CH, $^1J_{CP}$=154.04 Hz), 127.77 (*m*-C_{Ph}), 128.84 d (*o*-C_{Ph},$^4J_{CP}$= 1.10 Hz), 130.14 (p-C_{Ph}), 136.86 d (P-CH=C, $^2J_{CP}$= 3.65 Hz), 151.03 d (*ipso*-C_{Ph},$^3J_{CP}$ = 14.64 Hz). ^{31}P NMR spectrum, δ, ppm: 18.39.

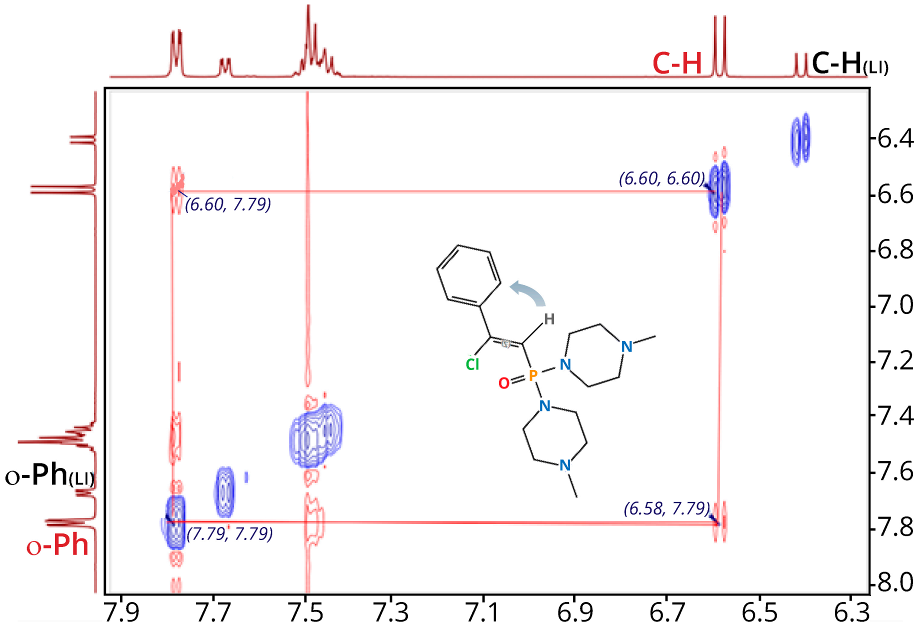

Fig. S1. NMR ^{1}H-^{1}H NOESY spectra of PP after 60 minutes of 266 nm laser irradiation.

FTIR spectra of PP before and after UV laser irradiation are presented in Fig.S3. To clearly observe the effect of laser irradiation, the difference in IR spectra between laser-irradiated and original PP was calculated. The main differences observed are listed in Table S1.

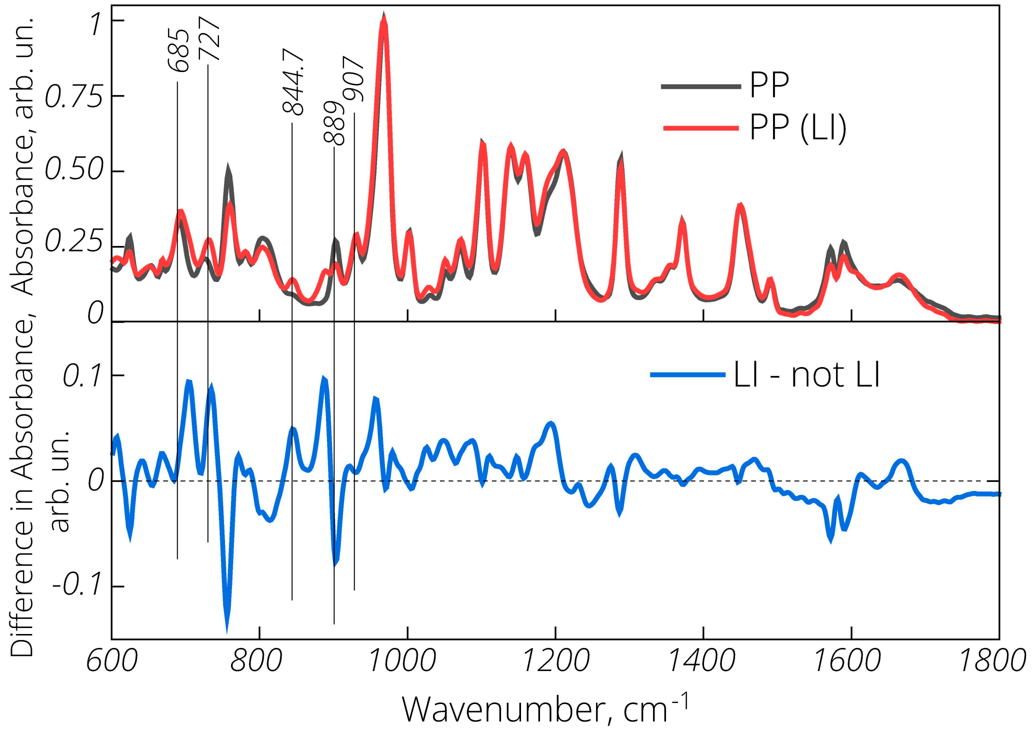

Fig. S2. IR absorption spectra of PP before and after UV laser irradiation.

Table S1. Selected IR absorption bands in the spectra of PP.

**Band position, cm^{-1}**	**The oscillations that cause the band**
685	1) Out-of-plane hydrogen vibrations out(HCCC) (originates from v11 in the benzene ring).

	2) Delocalized deformation vibrations, primarily δ (CCC) in benzene rings (resembling v6a vibrations in the benzene ring).
727	ν(N-P), δ(CNC), ρ(CH_2) in Ring_B.
844.7	Out-of-plane vibrations of hydrogen in the alkene part out(CPhCCH).
889	The terminal methylene group of =CH_2.
907	Mainly deformation vibrations in the alkene part δ(C=C-C), δ(C=C-P), as well as deformation vibrations in the benzene ring δ(CCC), originating from the v12 mode.

The presented data show that after irradiation of PP, new absorption bands with maxima at 844 and 889 cm^{-1} appear in its IR spectra. The band with a maximum at 889 cm^{-1} is caused by vibrations of the terminal methylene group. The appearance of this band and amplification of out-of-plane hydrogen vibrations in the alkene part (CPhCCH) after irradiation of PP correlate with cis-trans isomerization of the PP molecule during irradiation.

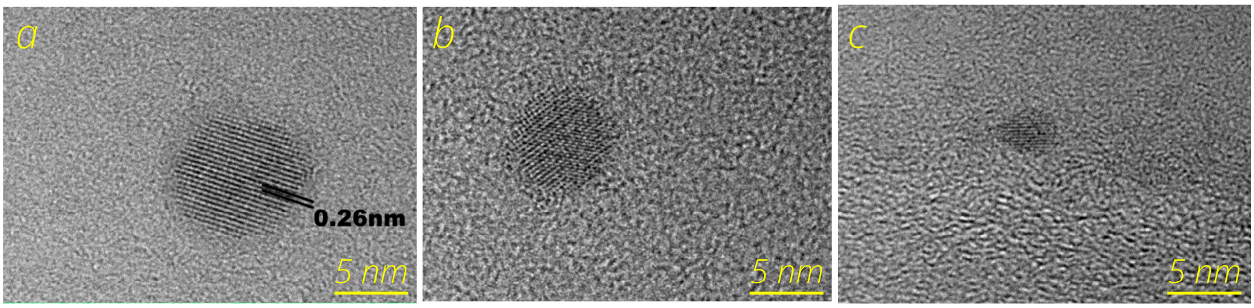

Fig. S3. HRTEM images of CQDs: (a) CQD1, (b) CQD2, (c) CQD3.

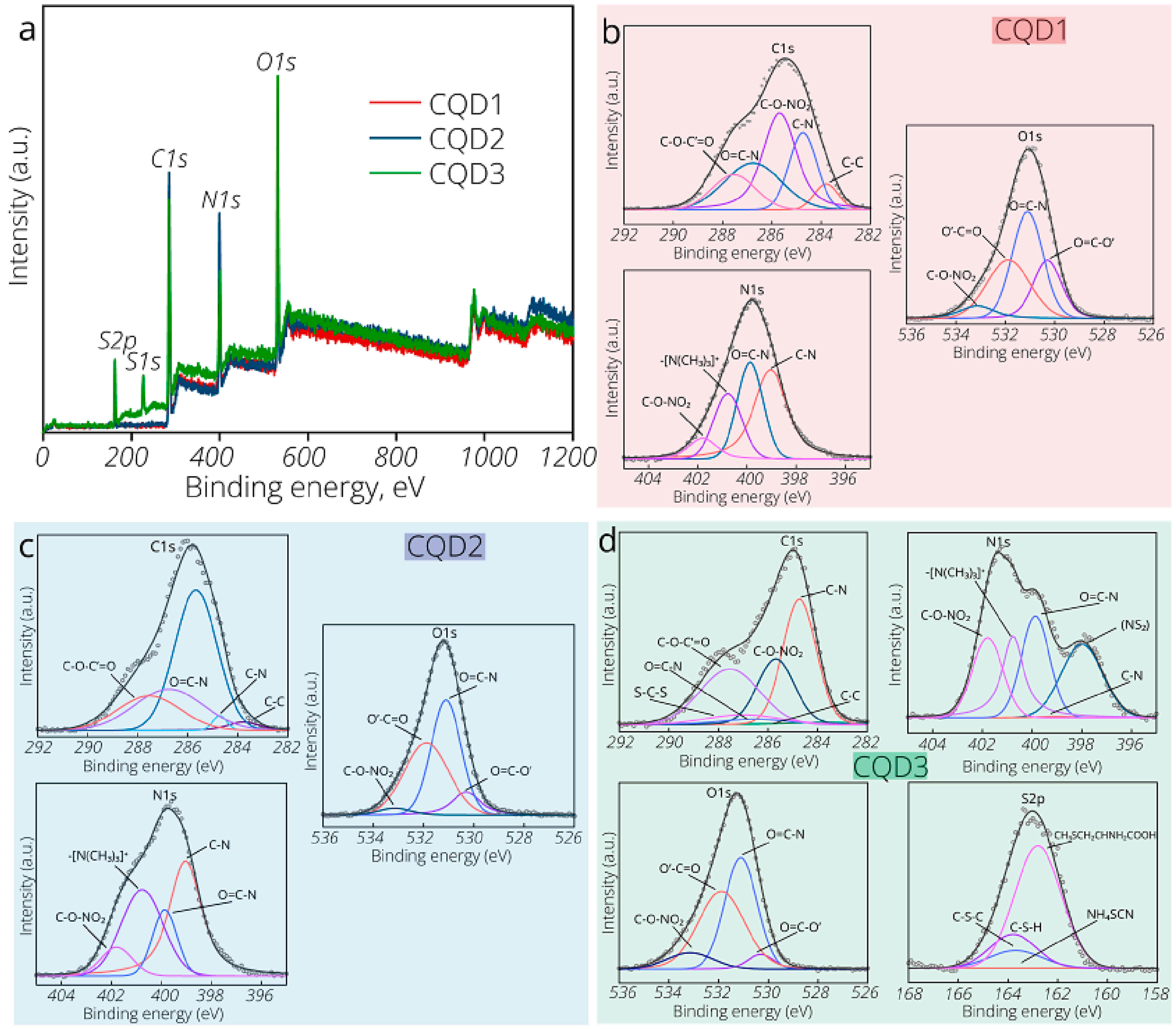

Fig. S4. (a) The XPS survey scans of the synthesized CQDs. (b)-(d) The XPS deconvoluted C1s, N1s, O1s, and S2p peaks of CQDs.

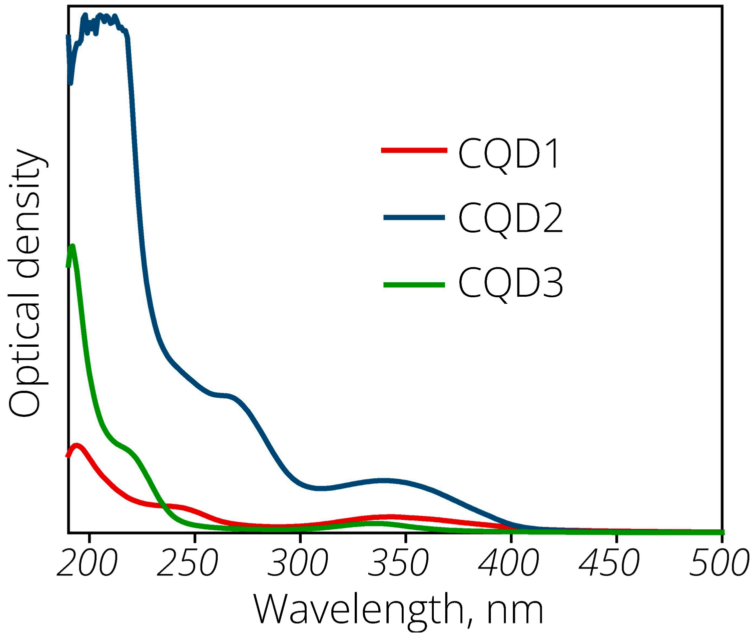

Fig. S5. Optical density spectra of aqueous solutions of CQD.

Detailed analysis of FTIR spectra of CQD1-3

In all CQDs' spectra, bands of vibrations of amide bonds (C=O)-N are observed, which are the result of the formation of a peptide bond between the carboxyl groups -COOH of CA and the amino groups $-NH_2$ of EDA or TU at the initial stage of synthesis. Further synthesis leads to a different composition of the functional groups of nanoparticles. Thus, CQD1 has predominantly (C=O)-N, -N=O, and $-CH_x$ groups on the surface with a small number of $-NO_2$ and -C≡N groups. CQD2 has predominantly (C=O)-N, $-NO_2$, -N=O, and $-CH_x$ groups on the surface. The spectrum of CQD2 also contains an intense absorption band at 1321 cm^{-1}, most likely due to C-N stretching, N-H bending and/or C-H deformation, and a band with a maximum at 1483 cm^{-1}, probably due to a combination of deformation and stretching vibrations of C-N, N-H, and C-H bonds. CQD3, doped with sulfur in addition to nitrogen, has a large number of -C≡N groups (probably in the thiocyanate group -S-C≡N), C-S bonds, (C=O)-N, and a small number of -N=O groups in its structure. In addition, the presence of C-O and C=O bond vibration bands indicates the presence of -COOH carboxyl groups in CQD3. However, the position of the C=O band at 1715 cm^{-1} (in contrast to the position at 1705 cm^{-1} for CA) indicates that -COOH is isolated from other carboxyl groups. Such a shift in the spectrum of CQD3 may also indicate that C-O and C=O are not in the same bond. Moreover, in all CQDs, -OH and -C=C- groups and bonds are possible, but in the obtained IR spectra, it is impossible to separate their absorption from the bands of water adsorbed on CQDs or from vibrations of amide groups (C=O)-N, respectively.

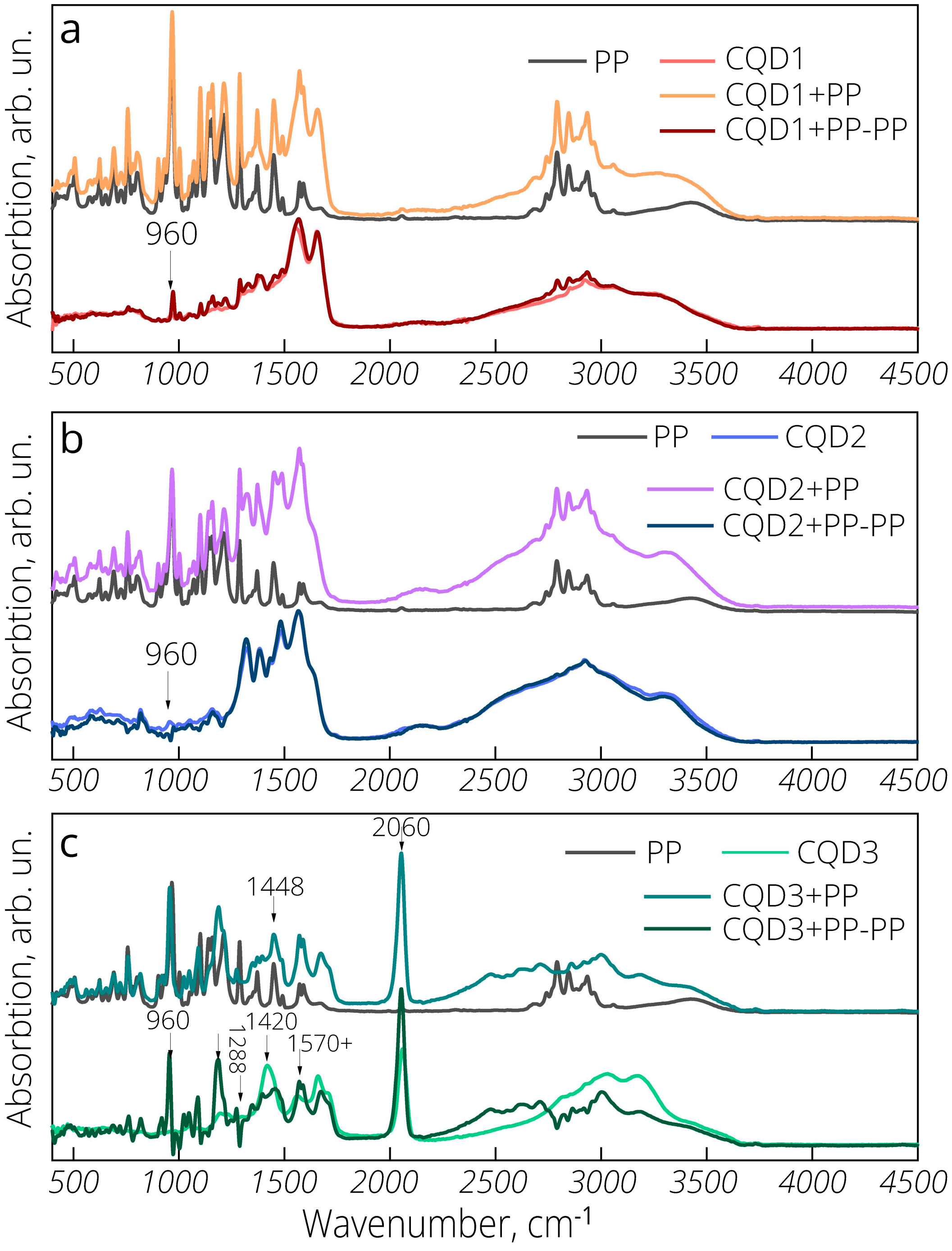

Fig. S6. FTIR spectra of individual CQDs, PP, and their mixtures (as obtained, not normalized, only offset) for (a) CQD1, (b) CQD2, and (c) CQD3. Spectra denoted as CQD+PP-PP are difference spectra between CQD+PP and PP, with PP spectra being adjusted to minimize the divergence between difference spectra and that of their CQDs.
